# Short-chain fatty acids inhibit bacterial plasmid transfer through conjugation *in vitro* and in *ex vivo* chicken tissue explants

**DOI:** 10.3389/fmicb.2024.1414401

**Published:** 2024-06-06

**Authors:** Logan C. Ott, Melha Mellata

**Affiliations:** ^1^Interdepartmental Microbiology Graduate Program, Iowa State University, Ames, IA, United States; ^2^Department of Food Science and Human Nutrition, Iowa State University, Ames, IA, United States

**Keywords:** horizontal gene transfer, short-chain fatty acids, plasmid transfer inhibition, antimicrobial resistance, bacterial plasmid conjugation

## Abstract

The animal gut acts as a potent reservoir for spreading and maintaining conjugative plasmids that confer antimicrobial resistance (AMR), fitness, and virulence attributes. Interventions that inhibit the continued emergence and expansion of AMR and virulent strains in agricultural and clinical environments are greatly desired. This study aims to determine the presence and efficacy of short-chain fatty acids (SCFA) inhibitory effects on the conjugal transfer of AMR plasmids. *In vitro* broth conjugations were conducted between donor *Escherichia coli* strains carrying AMP plasmids and the plasmid-less *Escherichia coli* HS-4 recipient strain. Conjugations were supplemented with ddH_2_O or SCFAs at 1, 0.1, 0.01, or 0.001 molar final concentration. The addition of SCFAs completely inhibited plasmid transfer at 1 and 0.1 molar and significantly (*p* < 0.05) reduced transfer at 0.01 molar, regardless of SCFA tested. In explant models for the chicken ceca, either ddH_2_O or a final concentration of 0.025 M SCFAs were supplemented to the explants infected with donor and recipient *E. coli*. In every SCFA tested, significant decreases in transconjugant populations compared to ddH_2_O-treated control samples were observed with minimal effects on donor and recipient populations. Finally, significant reductions in transconjugants for plasmids of each incompatibility type (IncP1ε, IncFIβ, and IncI1) tested were detected. This study demonstrates for the first time the broad inhibition ability of SCFAs on bacterial plasmid transfer and eliminates AMR with minimal effect on bacteria. Implementing interventions that increase the concentrations of SCFAs in the gut may be a viable method to reduce the risk, incidence, and rate of AMR emergence in agricultural and human environments.

## 1 Introduction

The emergence and spread of antimicrobial resistance (AMR) and virulence attributes in bacteria are of immense concern to both animal and human health (Teuber, 1999; Nordmann and Poirel, 2005; Robicsek et al., 2006; Sommer et al., 2009; Penders et al., 2013; Lopatkin et al., 2017; Fukuda et al., 2019; Hedman et al., 2020; Techitnutsarut and Chamchod, 2021). The gut of food animals, such as poultry, acts as a potent reservoir for this spread and storage of plasmids (Jochum et al., 2021). Plasmid spread among bacteria results in the emergence of novel bacterial strains with potential for pathogenicity and often results in resistant infections in both animal agriculture as well as in the clinical environments (Guiyoule et al., 2001; Wang et al., 2003; Nordmann and Poirel, 2005; Hong et al., 2009; Aguado-Urda et al., 2012; Soares et al., 2014; Tang et al., 2017; Dolejska and Papagiannitsis, 2018; Millan, 2018). In humans, this leads to prolonged care and recovery of patients, and increased mortality (Roca et al., 2015; Woolhouse et al., 2015; Hofer, 2019).

Identification of strategies for inhibiting conjugal spread of plasmids among bacteria in the environment and within animal hosts is of dire importance (Ruzin and Novick, 1998; Fernandez-Lopez et al., 2005; Ripoll-Rozada et al., 2016; García-Cazorla et al., 2018; Graf et al., 2018). The use of the diet has been briefly examined for its role in preventing AMR (Liu et al., 2019; Elshaghabee et al., 2021). Preliminary studies have demonstrated using medium and long-chain polyunsaturated fatty acids (PUFAs) to inhibit the conjugal ATPase TrwD. However, no *in vivo* or host-associated studies have confirmed this function, nor has the mechanism of action been confirmed (Fernandez-Lopez et al., 2005; Ripoll-Rozada et al., 2016; García-Cazorla et al., 2018). Regardless, a limitation to the application of PUFAs as a dietary intervention for gut-mediated plasmid transfer is that medium and long-chain fatty acids (MCFA, LCFA) are readily absorbed in the upper intestinal tract and often do not persist to the gut microbiota to mediate an effect *in vivo* (Ramírez et al., 2001).

Short-chain fatty acids (SCFAs) have a carbon chain length of no more than six and are potent regulators of the gut environment. Increased concentrations of SCFAs in the gut have been found to select for beneficial microbes, inhibit colonization and persistence of harmful microbes, and SCFAs act as a metabolic source for healthy epithelial cell growth and maintenance (Sakata and Yajima, 1984; Vinolo et al., 2011; Kim et al., 2014; Tan et al., 2014; Baxter et al., 2019; Parada Venegas et al., 2019; Schulthess et al., 2019; Liao et al., 2020). Contrary to MCFAs and LCFAs, SCFAs are the metabolic output of microbial fermentation of indigestible fibers from the host diet and are generated locally in the midgut by microbial constituents (Kim et al., 2014). Supplementation of the diet with nonfermentable fibers is currently understood to lead to increased gut homeostasis and epithelial health through the enrichment of SCFA and SCFA-producing bacteria (Baxter et al., 2019; Mueller et al., 2020). There is evidence for an inhibitory effect on the colonization and persistence of organisms commonly found to host plasmids through modification of the gut environment pH, but the combinatorial role of SCFA inhibition of both colonization and conjugation has not yet been demonstrated (Kim et al., 2014; Baxter et al., 2019; Schulthess et al., 2019; Liao et al., 2020).

Overall, the goal of this study is to determine what effect SCFAs have on the incidence of bacterial plasmid transfer in an *in vitro* model for the gut environment by testing the impact of the naturally occurring SCFAs from the animal gut, as well as the greater family of SCFAs to determine if the biochemical effects are dependent on fatty acid chain length. Additionally, we tested the broad efficacy of SCFAs against conjugal plasmids of varying genetic types to identify if inhibition is plasmid-specific or conserved across plasmid biology. Here, we demonstrate the apparent inhibition of bacterial plasmid conjugation in an *in vitro* and an *ex vivo* avian host co-culture ceca explant. Using traditional broth plasmid conjugation assays, we identified the ability of SCFAs to impact the rate and incidence of plasmid conjugation. Furthermore, we showed the ability of SCFAs to confer an effect in *ex vivo* host-associated environments, indicating a potential role *in vivo*.

## 2 Materials and methods

### 2.1 Chemicals and reagents

Unless otherwise stated, all SCFAs used in this study were purchased from VWR International (Radnor, Pennsylvania, United States). RPMI, ADMEM, fetal bovine serum, and L-glutamine were purchased from Thermo Fisher Scientific (Waltham, Massachusetts, United States) and were Gibco brand. SCFAs were dissolved and diluted into sterile High-performance liquid chromatography (HPLC) grade ultrapure water and filter sterilized through 0.22 μM syringe filters before use. SCFAs were stored covered at room temperature between uses. SafMannan^®^ yeast cell wall preparations were provided by Phileo by Lesaffre.

### 2.2 Bacterial strains and culture

The multidrug-resistant (MDR) Avian Pathogenic *Escherichia* (*E.*) *coli* (APEC) strain APEC O2-211 was used as the primary donor in both *in vitro* and *ex vivo* conjugation experiments (Nielsen et al., 2018). APEC harbors one large MDR plasmid, pAPECO2-211A-ColV (197 Kbp), and two small plasmids, pAPEC-O2-211B (4,231 bp) and pAPEC-O2-211C (2,096 bp). The pAPECO2-211A-ColV was the plasmid screened for transfer, and confers resistance to tetracycline, macrolide, fluoroquinolone, carbapenem, cephalosporin, and aminoglycoside antibiotics. Furthermore, pAPEC-O20211A-ColV contains the virulence determinants; *ibeA*, *iutA*, *iroN*, *sitA*, *tsh*, *fim*, *fyuA*, *pap*, and *vat* (Nielsen et al., 2018). *Salmonella* (*S*.) *enterica* Serovar Kentucky strain CVM29188 was isolated from a chicken meat sample and served as a supplemental donor during *in vitro* conjugation assays (30). CVM29188 contains 3 plasmids, 2 of which carry MDR, including pVCM29188_146 (146 kb, GenBank accession CP001122) that carries resistance genes to aminoglycosides and tetracyclines and pCVM29188_101 (101 kb, GenBank accession CP001121) that carries resistance to cephalosporins and quaternary ammonium compounds. For conjugations with the *S.* Kentucky strain, the transfer of the pCVM29188_146 plasmid was monitored as with the pAPEC-O2-211A-ColV plasmid.

To limit host effect on conjugation assays*, E. coli* SP915 strain, an MG1655 derivative with a chromosomal mCherry insertion, was used as the donor for the plasmids pKJK5-GM (tetracycline and trimethoprim resistant), pCVM29188_146 (tetracycline and streptomycin resistant), and pC20-GM (cefotaxime resistant) (Ott et al., 2021). The *E. coli* HS-4 strain maintained a spontaneous chromosomal resistance to rifampicin and was used as a recipient in all conjugations (Ott et al., 2020, 2021). *E. coli* HS-4 was initially isolated from a healthy human gut and was a proxy for a commensal animal gut microbe (Rasko et al., 2008). Full information on the strains and plasmids used in this study is reported in Table 1. Fresh bacterial cultures were streaked on MacConkey agar supplemented with antibiotics targeting each plasmid resistance marker (pAPEC-O2-211A-ColV, 15 μg/mL tetracycline; pKJK5-GM, 15 μg/mL tetracycline; pCVM29188_146, 15 μg/mL tetracycline; pC20-GM, 4 μg/mL cefotaxime; HS-4, 100 μg/mL rifampicin) before each experiment.

**Table 1:** Bacterial strains and plasmids.

Species	Strain	Role	Plasmid	Relevant properties	Origin
**E. coli**					
	APEC O2-211	Donor	pAPECO2-211A-ColV	IncFIβ/IncFIC, Tet^R^, Str^R^	Nielsen et al. (2018)
	MM0001	Donor	pKJK5-GM	SP915 transformant, Km^R^	Ott et al. (2021)
	MM0002	Donor	pCVM29188_146	SP915 transformant, Km^R^	Ott et al. (2021)
	MM0003	Donor	pC20-GM	SP915 transformant, Km^R^	Ott et al. (2021)
	SP915	Recipient	–	K-12 MG1655 lab strain; Km^r^	Popowska and Krawczyk-Balska (2013)
	HS-4	Recipient	–	Human commensal isolate, spontaneous rifampicin resistance	Ott et al. (2021)
**S. Kentucky**					
	CVM29188	Donor	pCVM29188_146 pCVM29188_101 pCVM29188_46	IncFIβ/IncFIC, Tet^R^, Str^R^ IncI1, *bla*_CMY-2_*, blc, sugE, cib* IncFII, *ccdAB*	Ott et al. (2020)
Plasmids		Replicon Type	Antimicrobial resistances	Other characteristics	Origin
**Narrow host range**					
	pAPECO2-211A-ColV	IncFIβ/IncFIC	Tet^R^, Str^R^, Azm^R^, Nov^R^, Cip^R^, Mem^R^, Mtz^R^	*cva*, *ibeA*, *iutA*, *iroN*, *sitA*, *tsh*, *fim*, *fyuA*, *pap*, *vat*	Nielsen et al. (2018)
	pCVM29188_146	IncFIβ	Tet^R^, Str^R^, Azm^R^, Nov^R^, Cip^R^, Mem^R^, Mtz^R^, Q-D^R^	*cva*, *cia*, *iutA*, *iro*, *sit*, *pap, sopAB, shiF, hlyF, ompT, ets*	Ott et al. (2020)
	pC20-GM	IncI1	Ctx^R^	[PA10403-gfpmut3]	Anjum et al. (2018)
**Broad host range**					
	pKJK5-GM	IncP-1ε	Tmp^R^, Tet^R^, Azu^R^	[PA10403-gfpmut3]	Popowska and Krawczyk-Balska (2013)

^R^, resistant; Km, kanamycin; Rif, rifampicin; Ctx, cefotaxime, Azm, Azithromycin; Mtz. Metronidazole; Nov, Novobiocin, Cip, Ciprofloxacin; Mem, Meropenem; Mtz, Metronidazole; Q-D, Quinupristin-Dalfopristin; Azu, Azulfidine.

### 2.3 *In vitro* conjugation assays

Conjugation reactions between donor strains and the recipient HS-4 strain were assayed through traditional broth conjugation methods using Luria Bertani (LB) broth as the suspension medium as previously (Ott et al., 2020, 2021). Briefly, overnight cultures of logarithmic phase donor and recipient strains were pelleted at 16,000 × G for 2 min, rinsed twice with pre-warmed phosphate-buffered saline (PBS), and the final pellet was then resuspended to OD_600nm_ ~ 1.0. Cultures were mixed 1:1 for a total volume of 180 μL in individual wells of a 96-well tissue culture plate. SCFAs were added at a dilution of 1:10 for final desired concentrations of either 0 and 0.025 M, or 0, 0.001, 0.01, 0.1, and 1 molar, or at physiological concentrations previously observed from the chicken ceca (Walugembe et al., 2015; González-Ortiz et al., 2020). Conjugation mixtures were then incubated for 6 h at the optimal bacterial growth temperature of 37°C. After incubation, conjugation mixtures were immediately serially diluted and plated on MacConkey agar selecting for each bacterial population (donors, 15 μg/mL tetracycline or 4 μg/mL cefotaxime; recipients, 100 μg/mL rifampicin; transconjugants, 15 μg/mL tetracycline or 4 μg/mL cefotaxime and 100 μg/mL rifampicin).

### 2.4 Chicken ceca explant preparation and *ex vivo* conjugation assay

All animal work in this study was reviewed and approved by the Institutional Animal Care Use Committee at Iowa State University under protocol IACUC-21-265. On two separate occasions, three ~2-week-old white leghorn chickens were collected from a local commercial hatchery, CO_2_ euthanized, and aseptically necropsied. From each bird, 3 cm long segments of each cecum were removed, opened longitudinally, and placed in tissue collection media (RPMI, 50 ug/mL gentamycin, 1% penicillin/streptomycin) and incubated at 40°C for 1 h to clear remaining resident microbiota and remove fecal material. Tissues were then rinsed in sterile PBS to remove residual antibiotics and any remaining fecal material, aseptically cut into 0.5 cm^2^ segments, and placed into individual wells of a 96-well tissue culture plate.

Complete growth media (ADMEM, 8 mM L-Glutamine, 1% Pen/strep, 10% fetal bovine serum) supplemented with OD_600nm_ ~ 1.0 of donor and recipient bacterial mixture prepared as previously described during *in vitro* conjugations *with complete growth media as the pellet diluent* was added. To each well of paired ceca tissue, sterile ddH_2_O or SCFA were added in 1:10 dilutions for a final concentration of 0.025 M and a final volume of 200 μL as to represent a realistic but increased concentration of SCFAs in the gut compared to physiological conditions. Following incubation at 40°C for 6 h in 5% CO_2_, supernatant from each tissue co-culture was pipette homogenized and serially diluted in tenfold dilutions and plated on MacConkey agar selecting for either donor (15 ug/mL tetracycline), recipient (100 ug/mL rifampicin), or transconjugants (15 ug/mL tetracycline and 100 ug/mL rifampicin) populations.

### 2.5 Statistical analysis and calculations

Statistical analyses were completed using the GraphPad Prism 6 Software suite. *In vitro,* ddH_2_O and SCFA groups were compared using unpaired two-way ANOVA (concentration ~ SCFA). *Ex vivo* ddH_2_O and SCFA groups were compared using a paired one-way ANOVA within each bacterial population. *p*-values (*p*) less than 0.05 were considered significant. When used, conjugation frequency was calculated as the total population of transconjugants divided by the total population of donors per sample.

## 3 Results

### 3.1 The SCFA propionate inhibits bacterial conjugation in a dose-dependent manner

**Figure 1:**
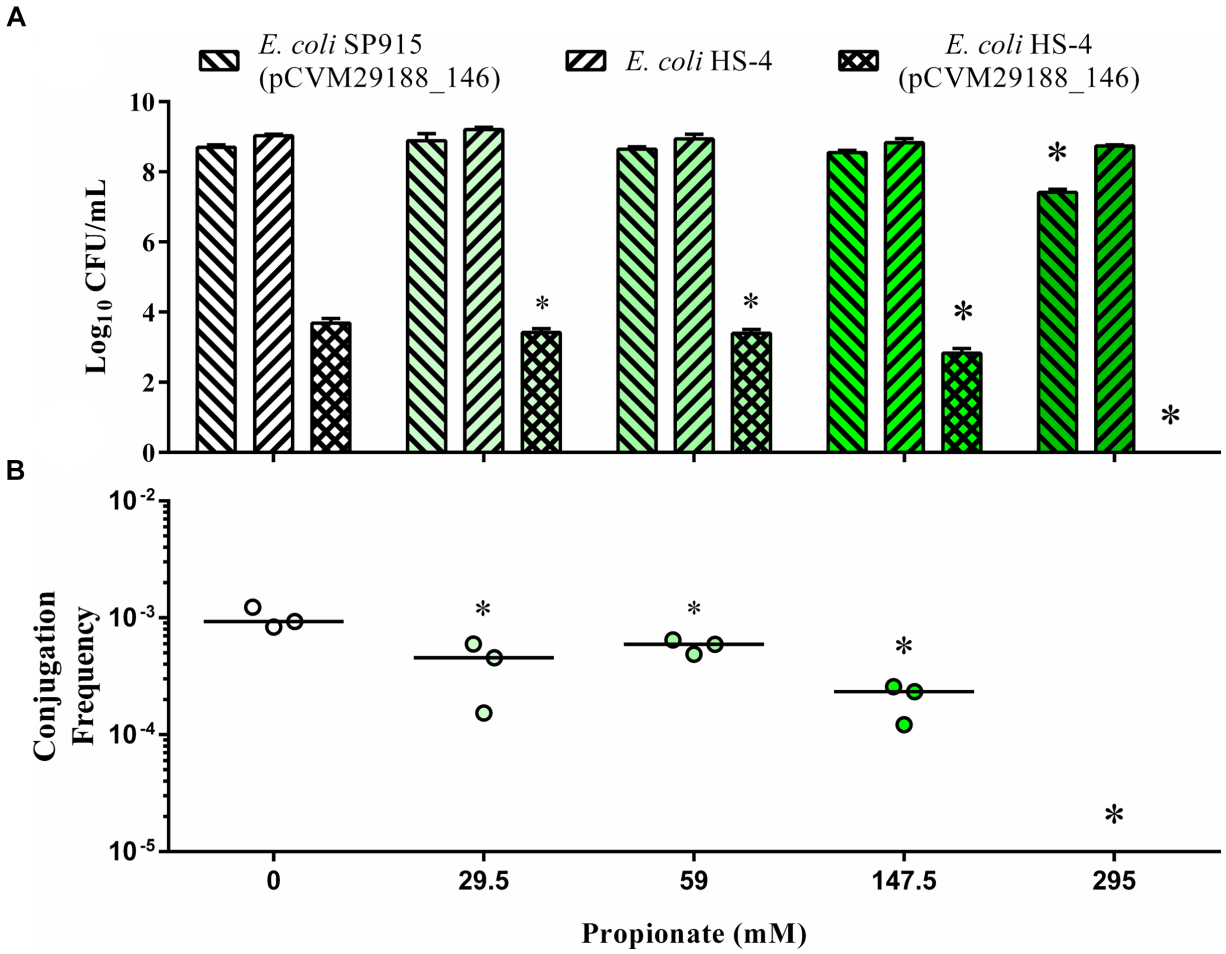
*In vitro* inhibition of bacterial plasmid transfer by the SCFA propionate in liquid broth. The Log_10_ CFU/mL of donors (forward slash), recipients (backward slash), and transconjugants (crossed) **(A)** and conjugation efficiency **(B)**, as calculated by the total transconjugant population divided by the total donor population for each reaction. Bars in **(A)** represent the mean and standard deviations, while the bars in **(B)** represent the median. *p*-values were corrected for multiple comparisons by Dunnet *post-hoc* hypothesis testing. *p < 0.05, *p < 0.005, *p < 0.0005, *p < 0.00005. *p*-values less than 0.05 were considered significant.

*In vitro* broth plasmid conjugations between *E. coli* SP915 (pCVM29188_146) and the plasmid-free *E. coli* HS-4 recipient resulted in significantly decreased transconjugants populations at all propionate treatment levels (29.5, 59, 147.5, and 295 mM), with no transconjugants detected at the 295 mM concentration following enrichment (Figure 1A). Donor populations in the 295 mM propionate treatment group were significantly reduced compared to the control group (Figure 1A). Additionally, the conjugation frequency was significantly decreased for all treatment groups compared to the control (Figure 1B).

### 3.2 The SCFAs acetate and butyrate at physiological concentrations of the chicken ceca significantly reduce conjugation

**Figure 2:**
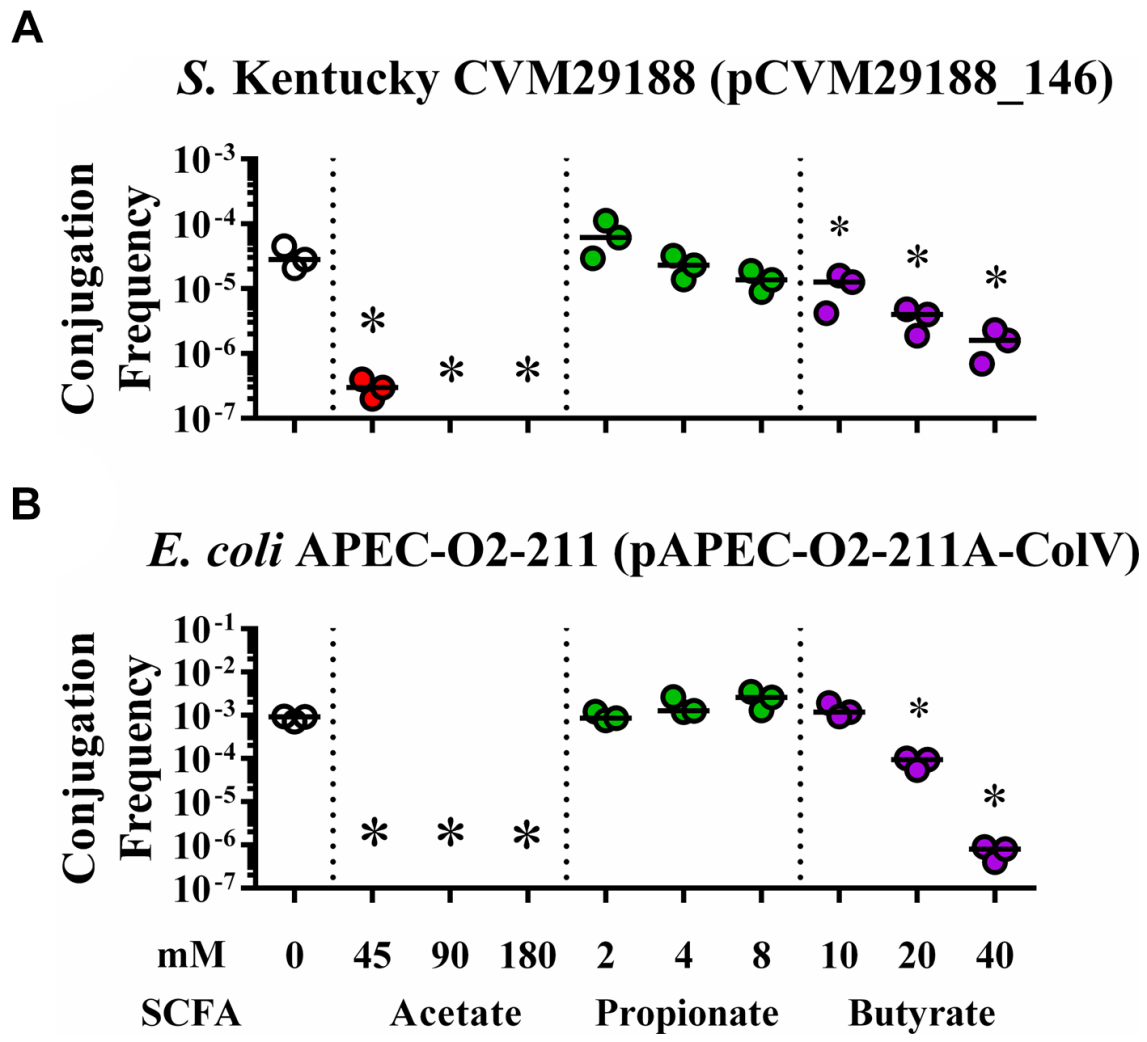
*In vitro* inhibition of bacterial plasmid transfer by SCFAs at gut physiological concentrations in liquid broth. Conjugation efficiency of plasmids pCVM29188_146 **(A)** and pAPEC-02–211A-ColV **(B)** during *in vitro* exposure to physiological levels of SCFAs from the chicken ceca. Bacteria were incubated with ddH_2_O (clear), acetate (red), propionate (green), or butyrate (purple) at 0, 45, 90, 180; 0, 2, 4, 8; and 0, 10, 20, or 40 mM, respectively. Bars represent the median. Comparisons between control and varying concentrations of SCFAs were completed by one-way ANOVA within each SCFA group. *P*s were corrected for multiple comparisons by Dunnet hypothesis testing. *p < 0.05, *p < 0.005, *p < 0.0005, *p < 0.00005. *p*-values less than 0.05 were considered significant.

Conjugation between either *S.* Kentucky or *E. coli* APEC-02–211 and the plasmid-free recipient *E. coli* HS-4 was demonstrated in the presence of acetate, propionate, and butyrate at physiological concentrations from the chicken ceca (Figure 2). Addition of acetate to the conjugation broths led to a significant reduction in conjugation frequency in all groups, with no detectable transconjugants in the 90 or 180 mM and 45, 90, and 180 mM groups for either *S.* Kentucky or APEC-02 (*p* < 0.05) (Figures 2A,B). Adding propionate at 2-, 4-, and 8-mM did not result in significant changes in conjugation frequency. However, a negative trend is visible in the *S.* Kentucky group, and a positive trend is present in the APEC-O2 group (*p* > 0.05) (Figures 2A,B). Addition of butyrate at 10, 20, or 40 mM demonstrated significant reductions in both donor groups in a dose-dependent response (*p* < 0.005) except for 10 mM in the APEC-02 group (*p* > 0.05) (Figures 2A,B).

### 3.3 Dose-dependent reduction in transconjugant populations in the presence of SCFAs

**Figure 3:**
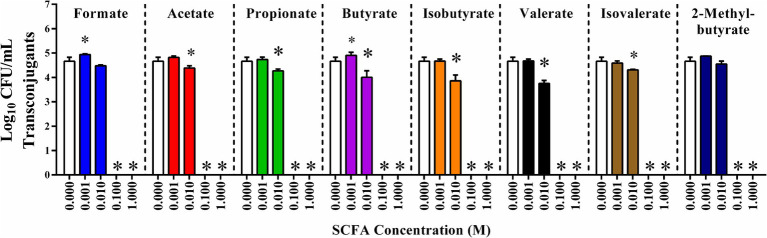
*In vitro* inhibition of IncF plasmid transfer by SCFAs in liquid broth. Conjugation reaction mixture supplemented with 0 (ddH_2_O), or 0.001, 0.01, 0.1, or 1 molar of each SCFA: formate (light blue), acetate (red), propionate (green), butyrate (purple), isobutyrate (orange), valerate (black), isovalerate (brown), and 2-methylbutyrate (dark blue). Bars represent the mean of three replicates, and error bars represent the standard deviation above and below the mean. Comparisons between ddH_2_O treatments and varying concentrations of SCFAs were completed by one-way ANOVA within each SCFA group. *P*s were corrected for multiple comparisons by Dunnet hypothesis testing. *p < 0.05, *p < 0.005, *p < 0.0005, *p < 0.00005. *p*-values less than 0.05 were considered significant.

To explore the role of SCFAs on the incidence of plasmid conjugation, liquid *in vitro* plasmid conjugation assays between *E. coli* APEC-02–211 and *E. coli* HS-4 were conducted in the presence of 0, 0.001, 0.01, 0.1, and 1 M of either formate, acetate, propionate, butyrate, isobutyrate, valerate, isovalerate, or 2-methyl butyrate (Figure 3). Additions of each SCFA at 0.1 M or 1 M resulted in complete reductions in transconjugant populations, with no transconjugants detected following enrichment (*p* < 0.00005). Additionally, 0.01 M concentrations of either acetate, propionate, butyrate, valerate, or isovalerate resulted in significant decreases in transconjugant populations compared to the ddH_2_O treatment control (*p* < 0.005) (Figure 3). The 0.001 mM of both formate and butyrate treatments resulted in small but significant increases in total transconjugant populations (*p* < 0.05). No other significant differences between the 0.001 M SCFAs and the control were observed.

### 3.4 The addition of each of the eight SCFAs tested to coculture explant conjugations results in a significant decrease in transconjugant populations

**Figure 4:**
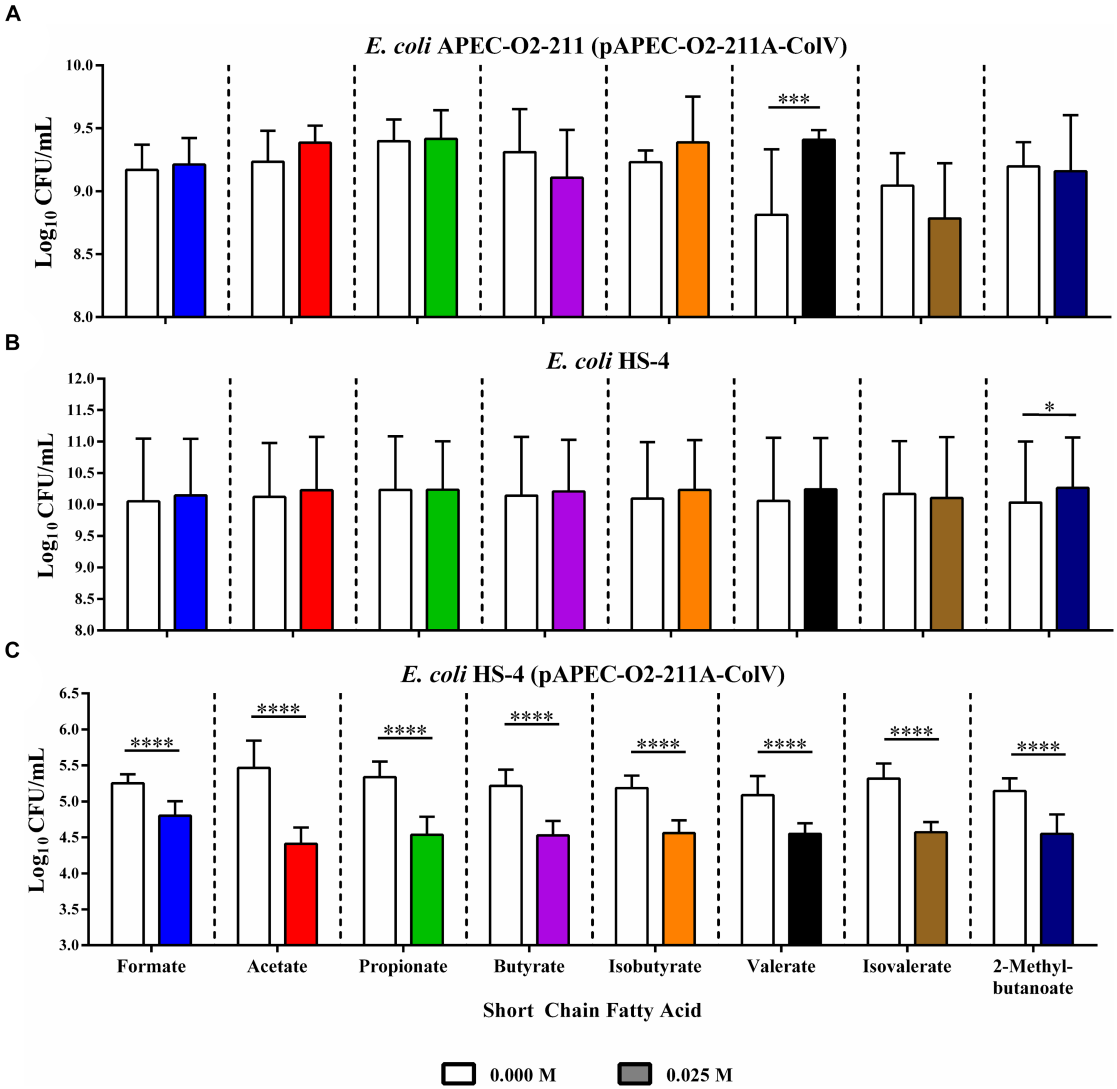
*Ex vivo* inhibition of IncF conjugation by SCFAs in chicken cecal explants. Bacterial enumeration of donors **(A)**, recipients **(B)**, and transconjugants **(C)** from cecal explant co-cultures. Bars represent the mean of three replicates, and error bars represent the standard deviation above and below the mean. Comparisons between ddH_2_O and SCFA treatments were completed by paired two-way ANOVA, and the Šidák correction for multiple comparisons was applied. *p < 0.05, *p < 0.005, *p < 0.0005, *p < 0.00005. *p*-values less than 0.05 were considered significant.

Chicken ceca explants were harvested and used in co-culture conjugation experiments to determine if the reduction in conjugation was observable in host-associated environments. The total donor (Figure 4A), recipient (Figure 4B), and transconjugant populations (Figure 4C) were, respectively, enumerated from co-cultures in the presence or absence of supplemental SCFAs at physiological concentrations (Figure 4). The addition of SCFAs individually to explant cultures resulted in slight but insignificant increases in donor APEC-02-211 populations in the formate, acetate, propionate, and isobutyrate treatment groups (Figure 4A). However, the increase in the donor population in the valerate group compared to the ddH_2_O control was significant (*p* < 0.0005). A non-significant decrease in the donor population was further observed in the butyrate, isovalerate, and 2-methylbutyrate groups (Figure 4A).

Likewise, the recipient populations were slightly, but insignificantly, increased in formate, acetate, butyrate, isobutyrate, valerate, and 2-methyl butyrate treatment groups, with the increase in recipient population in the 2-methyl butyrate treatment group being statistically significant (*p* < 0.05) (Figure 4B). The addition of isovalerate resulted in a slight, but insignificant, decrease in the total recipient population.

Finally, the addition of any of the eight SCFAs at 0.025 mM resulted in a large and significant reduction in total transconjugant populations when compared to the corresponding ddH_2_O-treated control groups (*p* < 0.00005) (Figure 4C).

### 3.5 Inhibition of plasmid conjugation by SCFAs at physiological concentrations occurs on a plasmid-type independent basis

**Figure 5:**
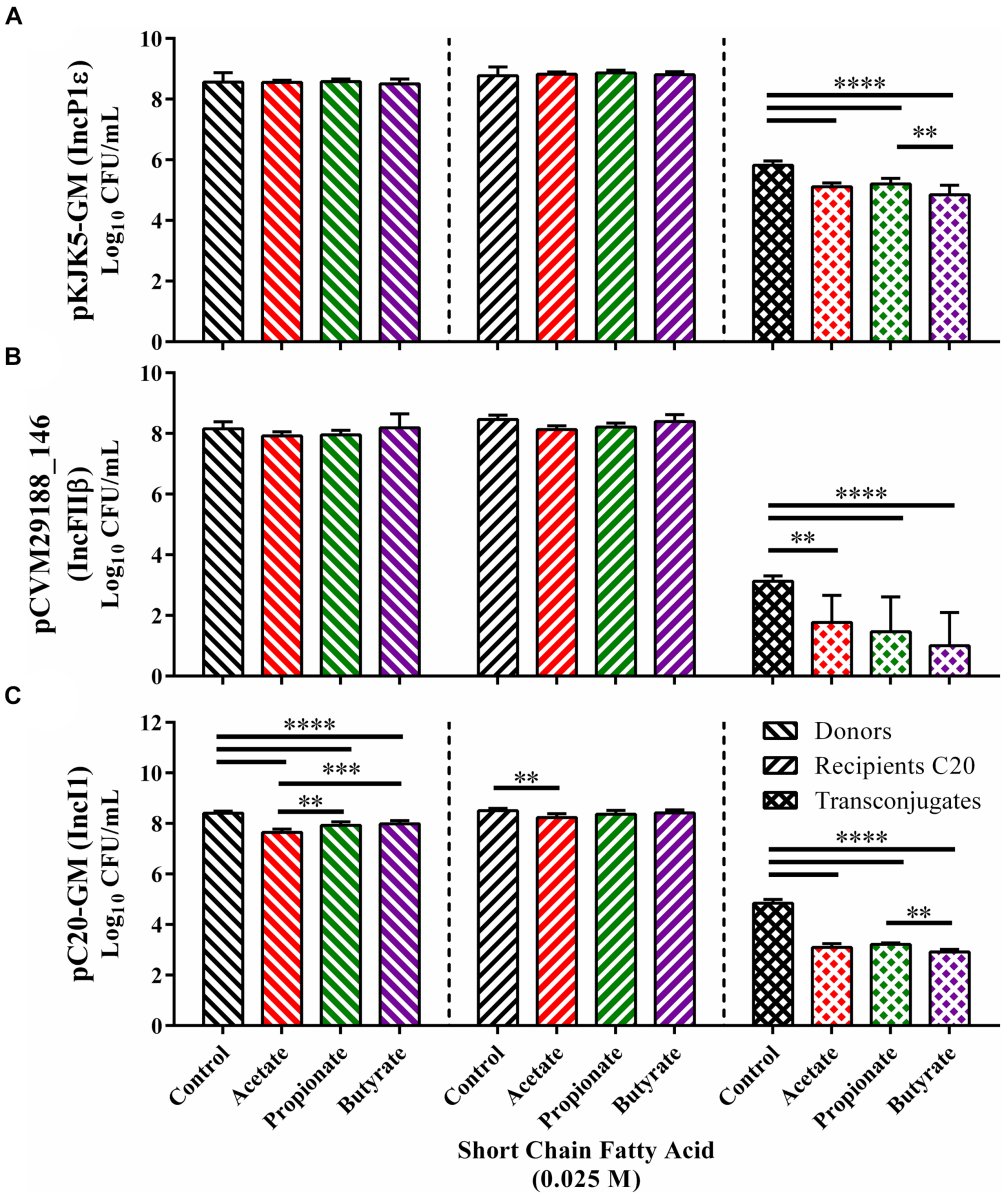
*In vitro* inhibition of the transfer of multiple plasmid incompatibility groups in liquid broth. Conjugation in the presence of acetate (red), propionate (green), and butyrate (purple) for *E. coli* SP915 (pCVM29188_146) **(A)**, *E. coli* SP915 (pKJK5-GM) **(B)**, and *E. coli* SP915 (pC20-GM) **(C)**. Comparisons between ddH_2_O and SCFA treatments were completed by paired two-way ANOVA, and the Šidák correction for multiple comparisons was applied. *p < 0.05, **p < 0.005, ***p < 0.0005, ****p < 0.00005. *p*-values less than 0.05 were considered significant.

To ascertain whether the inhibitive properties of SCFAs occurred consistently with plasmids of various incompatibility types, *in vitro* conjugations between *E. coli* SP915 carrying either pKJK5-GM (IncP1ε), pCVM29188_146 (IncFIβ), or pC20-GM (IncI1) and the plasmid-less HS-4 recipient were conducted in the presence or absence of 0.025 M of the gut associated SCFAs acetate, propionate, or butyrate (Figure 5). The addition of each of the SCFAs tested to conjugations between *E. coli* SP915 containing the pKJK5-GM broad host range plasmid did not result in significant changes in donor or recipient populations compared to the ddH_2_O treated control group (Figure 5A). However, total transconjugant populations were significantly decreased (*p* < 0.00005) in each of the SCFA treatment groups when compared to the ddH_2_O treatment control group. Furthermore, the butyrate treatment resulted in significantly fewer transconjugants compared to the propionate (*p* < 0.005) but not compared to the acetate.

In plasmids conjugations between *E. coli* SP915 carrying the pCVM29188_146 narrow host range plasmid and HS-4, again, no significant differences between donor and recipient populations and the ddH_2_O treated controls were observed (Figure 5B). However, significant reductions in the transconjugant populations were observed in all three SCFA groups tested with the most significant decreases being in propionate and butyrate (*p* < 0.00005) and a lesser but significant (*p* < 005) reduction in the acetate treatment group when compared to the ddH_2_O treatment group (Figure 5B).

Finally, plasmid conjugations between *E. coli* SP915 carrying the narrow host range plasmid pC20-GM and the plasmid-free HS-4 resulted in significant (*p* < 0.00005) reductions in donors for each of the three SCFAs acetate, propionate, butyrate tested with decreasing reductions from acetate to propionate (*p* < 0.005), and acetate to butyrate (*p* < 0.0005) (Figure 5C). Additionally, a significant decrease in recipients was observed in the acetate group but not in the propionate nor butyrate treatment groups. Again, total transconjugant populations were significantly decreased (*p* < 0.00005) in each SCFA treatment group compared to the ddH_2_O treatment control group. Furthermore, the butyrate treatment group resulted in significantly fewer transconjugants compared to propionate (*p* < 0.005) but not compared to acetate (Figure 5C).

## 4 Discussion

The misuse of antibiotics in both agricultural and clinical settings has resulted in the rapid evolution and dissemination of AMR and virulence genes (Threlfall et al., 2000; Lowy, 2003; Nordmann and Poirel, 2005; Robicsek et al., 2006; Roca et al., 2015). This dissemination is empowered through the use and transfer of extrachromosomal plasmid DNA (Dolejska and Papagiannitsis, 2018; Millan, 2018; Redondo-Salvo et al., 2020). The transfer of this DNA through bacterial conjugation enables the direct transfer and rapid expression of whole AMR and virulence genes (Licht et al., 1999; Grohmann et al., 2003; Mellata et al., 2010; Virolle et al., 2020). This phenomenon contributes to the rapid emergence and spread of novel MDR pathogens not only in human healthcare but also in agricultural environments (Dowson et al., 1989; Guiyoule et al., 2001; Wang et al., 2003; Skyberg et al., 2006; Hong et al., 2009; Mellata et al., 2010, 2012; Aguado-Urda et al., 2012; Soares et al., 2014; Card et al., 2017; Lacey et al., 2017; Millan, 2018; Mobasseri et al., 2019). This is significant due to the transfer of bacterial pathogens from food products, such as poultry meat, to humans having been frequently observed (Fricke et al., 2009; Mellata, 2013; Mitchell et al., 2015; Liu et al., 2018; Mellata et al., 2018).

With the emergence and spread of MDR plasmids, a new emphasis on non-antibiotic therapeutics for managing the microbiome associated with humans and animals has been developed (Fernandez-Lopez et al., 2005; Solis-Cruz et al., 2020; Cabezn et al., 2017; Lopatkin et al., 2017; Zhang et al., 2019; Li et al., 2021). Inhibition of bacterial plasmid transfer, as opposed to indiscriminately eliminating bacterial hosts, is a desirable approach to preventing the spread of MDR plasmids. This targeted approach avoids directly remodeling the gut microbiota through the depletion of potential reservoir strains such as the recipient populations. The resident strains are often beneficial or neutral in the gut and act as an alternate or temporary host for plasmid DNA (Salyers et al., 2004; Huddleston, 2014; Lerner et al., 2017). A plasmid specific approach is different from using antigen-based vaccination or even the use of antibiotics themselves, both of which remodel the gut microbiota by both discriminatory and nondiscriminatory depletion of microbiota members (Midtvedt et al., 1986; Candon et al., 2015; Mir et al., 2019; Zhang et al., 2019; Guevarra et al., 2021; Techitnutsarut and Chamchod, 2021). Alternative approaches to address this shift in focus have been regularly reported and examined for their ability to regulate the microbial populations in these environments and are periodically reviewed (Cohen, 2009; McDougall et al., 2009; Ghouri et al., 2018; Du et al., 2021; Sadowska et al., 2021; Wisener et al., 2021).

On such approach involves the use of dietary additives, such as the PUFAs linoleic acid and alpha-linolenic acid, which have been briefly demonstrated as conjugation inhibitors (COINs) (Li et al., 2021). However, the application of this novel dietary intervention has not yet been shown in host-associated conditions. Additionally, using medium to long-chain PUFAs offers limitations for practical use, as in the animal host, they are rapidly assimilated in the small intestine (Krogdahl, 1985; Ramírez et al., 2001). An alternative to the use of medium to long-chain PUFAs is the use of the microbial secreted SCFA dietary metabolites, as they are generated by the gut resident microbiota in the caecum of fermentative animals and in the colon of non-fermentative animals such as humans (Tan et al., 2014). The SCFAs have potent effects on the host gut and gut microbiota that are categorized as beneficial (Sakata and Yajima, 1984; Vinolo et al., 2011; Kim et al., 2014; Tan et al., 2014; Baxter et al., 2019; Parada Venegas et al., 2019; Liao et al., 2020). Unlike medium to long-chain PUFA, SCFAs escape rapid absorption in the small intestine as non-digestible intermediates, making them ideal candidates for directed regulation of the microbiota in the context of the gut (Krogdahl, 1985; Ramírez et al., 2001).

Propionate, a SCFA with a three-carbon chain length, is used as an antifungal and bacteriostatic preservative in foods, such as in bread and cheese, as well as in technical applications, such as in the media recipes used for the maintenance of *Drosophila* (Suhr et al., 2004; Guynot et al., 2005; Marin et al., 2010; BDSC, 2019). Additionally, propionate is one of the three SCFAs regularly found in the gut (i.e., acetate, propionate, and butyrate). Propionate is currently proposed to act on fungi by crossing the cell wall and interacting with succinyl-CoA, an intermediate of the metabolite catabolism pathway, to form propionyl-CoA at significantly increased levels. Subsequently, increased concentrations of propionyl-CoA inhibit the catabolism or anabolism of metabolites and prevent metabolic activity in fungi (Brock et al., 2000).

Initial conjugations with SCFAs were conducted as part of our previous work with the *Drosophila* animal model and included concentrations typical of those found in *Drosophila* media (Figure 1) (Ott et al., 2021). The minimal concentration for antimicrobial and antifungal effects of propionate have been previously studied and are inherently not novel (Maruyama and Kitamura, 1985; Brock et al., 2000; Guynot et al., 2005; Marin et al., 2010; Langfeld et al., 2021). Minimal inhibitory concentrations of propionate on *E. coli* and fungal growth were reported to be ~25 and 50 mM, respectively, levels which were similar to or lower than those found in *Drosophila* media (59 mM) (Brock et al., 2000; Guynot et al., 2005; Langfeld et al., 2021). While the concentrations we used for our initial *in vitro* conjugations were similar in scale, they were still greater than those traditionally identified in the gut of humans and other animals under physiological conditions (Sakata and Yajima, 1984; Liao et al., 2020; Mueller et al., 2020).

Unlike in fungi, the currently proposed mechanism of action of propionate in bacteria is like that of PUFAs, where medium-chain PUFAs are expected to bind to and directly inhibit the TrwD ATPase protein itself (Ripoll-Rozada et al., 2016; García-Cazorla et al., 2018). The contribution of the carboxylic acid functional group may be responsible for the conserved inhibitory effects of both SCFAs and longer-chain PUFAs in inhibiting bacterial plasmid conjugation. Structural analysis of the binding of SCFAs to conjugal proteins is also underway to determine if that mechanism may also provide a combinatory effect here. However, there is additional evidence that the import of propionate occurs in a protonated form and is rapidly deprotonated upon entry into the cell, increasing cytoplasmic pH and significantly increasing proton concentration (Maruyama and Kitamura, 1985; Langfeld et al., 2021). The increase in intracellular hydrogen ions may be critical to inhibiting the use of the proton gradient in producing ATP through membrane-bound ATP synthase (Langfeld et al., 2021). Inhibition of ATP synthase using the proton gradient may be essential in the functional transfer of plasmid DNA *in vitro* and even *in vivo,* as plasmid DNA processing, loading, and transfer are all dependent on the hydrolysis of cytoplasmic ATP (Willetts and Wilkins, 1984). However, the increase in cytoplasmic pH and the effect on the incidence or rate of plasmid conjugation are yet to be determined for SCFAs.

Poultry may be a primary source of MDR bacteria, and the transfer of such bacteria to humans (Al-Ghamdi et al., 1999; van den Bogaard et al., 2001; Fricke et al., 2009; Bortolaia et al., 2016; Hedman et al., 2020; Jochum et al., 2021). In this study, we first tested *in vitro* bacterial conjugations using the physiological concentration of SCFAs from the chicken ceca for the three main gut SCFAs, including acetate, propionate, and butyrate (Figure 2). We observed significant reductions in the frequency of plasmid conjugation for two MDR plasmids with significance to the poultry industry (pCVM29188_146 and pAPEC_02–211A-ColV). However, in these conjugations, the physiological concentration of propionate does not appear to be great enough to significantly reduce conjugation frequency as it is for acetate or butyrate. This indicates that at physiological levels of 2, 4, and 8 mM, propionate does not play a significant role in regulating conjugation in broth and likely does not at typical levels within the ceca of chickens (Dunkley et al., 2007; Józefiak et al., 2010; Sunkara et al., 2011). Acetate and butyrate, conversely, significantly reduced conjugation *in vitro,* as indicated by the significant reduction in conjugation frequency at all concentrations tested (Figure 2).

While the gut SCFAs acetate, propionate, and butyrate may have potential as COINs, it is not immediately clear if this is a conserved characteristic of all SCFAs or limited to those that regularly occur in the gut. To address this uncertainty, we repeated *in vitro* conjugations in the presence of all eight standard SCFAs at concentrations ranging between 1 and 0.001 M final concentrations (Figure 3). The complete depletion of transconjugant populations for all SCFAs at concentrations of SCFA of 0.1 and 1 M may be attributed to the intense shift in the pH of the media. It may make these extreme concentrations impractical or physiologically incompatible with the gut environment, so these specific results may not reflect the practical application of these concentrations *in vivo*.

We did, however, observe a significant reduction in the total transconjugant population in six of the eight SCFAs tested: acetate, propionate, butyrate, valerate, and isovalerate, with a trending decrease in the other two, formate and 2-methyl butyrate at a final concentration of 0.01 M. This concentration is partially between the previously reported minimal growth inhibition value and the physiological concentrations previously tested and found in the cecum of chickens (Dunkley et al., 2007; Józefiak et al., 2010; Sunkara et al., 2011). These results indicate that the greater family of SCFAs share a physiochemical factor that mediates the inhibitory effect observed in these studies, and further experimentation is required to identify and potentially optimize this approach. Furthermore, these data indicate that elevating the physiological concentrations of SCFAs in the gut may be a practical way to increase the observed reduction in transconjugants *in vivo*.

To mitigate the complexities of the *in vivo* gut environment and to overcome the limitations of *in vitro* models’ ability to account for complex host factors, we repeated conjugation assays in a chicken ceca explant model (Ott and Mellata, 2022). Chicken ceca explants were collected from two-week-old chickens from a commercial farm and used as substrate in broth conjugation reactions using donor and recipient populations in the presence of ddH_2_O or 0.025 M of each SCFA (Figure 4). To eliminate the possibility that pH changes due to the addition of SCFAs, not SCFAs themselves, may have caused the significant changes observed in our experiments, *ex vivo* explant conjugations were completed in a pH buffered solution, which mitigates changes in pH.

Similarly to our *in vitro* data, we observed little to no effect on the donor and recipient populations for each SCFA tested, with the exemption of a small but significant increase in donors in the valerate treatment group, and a small but significant rise in recipients in the 2-methylbutyrate treatment group. This data indicates that each of the eight SCFAs may have potential as a COIN in host-associated environments. Our data warrant future follow up *in vivo* studies to further examine the role of SCFA in regulating the incidence and rate of bacterial plasmid transfer in the gut of living hosts with intact resident microbiota.

In addition to the role of host association in microbial activity, the biology of bacterial plasmid transfer also relies on the plasmids’ genetic content (Gama et al., 2017; Virolle et al., 2020; Ott et al., 2021). As for the plasmids used in this study, each encodes their own transfer machinery, enabling them to be individually sufficient for both replication and transfer within the donor cell. However, the specific proteins and genes involved in the replication and transfer of each plasmid vary (Shintani et al., 2015). The genetic differences dictate the breadth of possible conjugal recipients and may lead to differential responses to COINs. To determine if SCFAs offer a COIN effect on a plasmid specific basis, we tested three plasmids of both broad and narrow host range types for conjugation under exposure to SCFAs. We observed a universal reduction in total transconjugant populations for each plasmid type tested *in vitro* (IncP1ε, IncFIβ, and IncI1) (Figure 5). These data indicate that SCFAs do have a universal mechanism of action for inhibiting plasmid conjugation outside of targeting specific genes. This may be different than what was previously observed with medium-chain PUFAs or may be additive and provide a synergistic outcome (Li et al., 2021).

Our study models the transfer of both broad and narrow host range MDR plasmids from both pathogenic and commensal *E. coli* to a representative commensal *E. coli* from the human gut, which then served as both a reservoir and potential pathobiont strain with potential virulence and pathogenic capacity (Salyers et al., 2004; Sommer et al., 2009; Penders et al., 2013; Rolain, 2013). The IncP pKJK5 plasmid is a MDR plasmid that maintains determinants of resistance to both tetracycline and sulfasalazine class antibiotics (Table 1) (Klümper et al., 2015). Likewise, both pCVM29188_146 and pAPEC-O2-211A-ColV are narrow host range (IncF) plasmids that maintain multiple AMR and virulence genes and are associated with food production hosts, such as chickens (Table 1) (Fricke et al., 2009; Nielsen et al., 2018). The ability of AMR and virulent plasmids from broad host range, like IncP plasmids, to transfer to a wide range of recipient organisms, including Gram-positive and Gram-negative bacteria of multiple genera and species (Klümper et al., 2015), could lead to diverse severe infections and antibiotic treatment failure. Thus, treatment like ours using SCFAs that significantly reduce and even eliminate plasmid transfer would highly benefit human health by preventing complications from infections and even death.

Overall, this study demonstrates SCFAs as a potential universal conjugation inhibitor during *in vitro* broth conjugations and chicken ceca explant co-culture experiments. While this does not directly demonstrate the *in vivo* action of SCFAs as COINs, it may hint at their role in the gut as COINs and identify them as potential novel therapeutic or dietary interventions to mitigate the emergence and spread of MDR strains in the gut. Continued studies on both the complete mechanism of action of SCFA COIN and *in vivo* application of SCFAs are ongoing.

## Data availability statement

The raw data supporting the conclusions of this article will be made available by the authors, without undue reservation.

## Ethics statement

The animal study was approved by Iowa State University, Institutional Animal Care Use Committee Protocol IACUC-21-265. The study was conducted in accordance with the local legislation and institutional requirements.

## Author contributions

LCO: Conceptualization, Data curation, Formal analysis, Funding acquisition, Investigation, Methodology, Visualization, Writing – original draft, Writing – review & editing. MM: Conceptualization, Formal analysis, Funding acquisition, Methodology, Project administration, Resources, Supervision, Validation, Writing – original draft, Writing – review & editing.
